# Integrated Proteomics and Metabolomics of Arabidopsis Acclimation to Gene-Dosage Dependent Perturbation of Isopropylmalate Dehydrogenases

**DOI:** 10.1371/journal.pone.0057118

**Published:** 2013-03-22

**Authors:** Yan He, Shaojun Dai, Craig P. Dufresne, Ning Zhu, Qiuying Pang, Sixue Chen

**Affiliations:** 1 Department of Biology, Genetics Institute, and Plant Molecular & Cellular Biology Program, University of Florida, Gainesville, Florida, United States of America; 2 Alkali Soil Natural Environmental Science Center, Northeast Forestry University, Key Laboratory of Saline-alkali Vegetation Ecology Restoration in Oil Field, Ministry of Education, Harbin, Heilongjiang, China; 3 Unity Lab Services, Thermo Fisher Scientific Inc., West Palm Beach, Florida, United States of America; 4 Interdisciplinary Center for Biotechnology Research, University of Florida, Gainesville, Florida, United States of America; Laurentian University, Canada

## Abstract

Maintaining metabolic homeostasis is critical for plant growth and development. Here we report proteome and metabolome changes when the metabolic homeostasis is perturbed due to gene-dosage dependent mutation of *Arabidopsis* isopropylmalate dehydrogenases (*IPMDHs*). By integrating complementary quantitative proteomics and metabolomics approaches, we discovered that gradual ablation of the oxidative decarboxylation step in leucine biosynthesis caused imbalance of amino acid homeostasis, redox changes and oxidative stress, increased protein synthesis, as well as a decline in photosynthesis, which led to rearrangement of central metabolism and growth retardation. Disruption of *IPMDHs* involved in aliphatic glucosinolate biosynthesis led to synchronized increase of both upstream and downstream biosynthetic enzymes, and concomitant repression of the degradation pathway, indicating metabolic regulatory mechanisms in controlling glucosinolate biosynthesis.

## Introduction

Amino acid homeostasis is pivotal for plant growth and development. Among the essential amino acids, leucine (Leu), valine (Val) and isoleucine (Ile) constitute a small group of branched-chain amino acids (BCAAs). In the past years, extensive research in the biosynthesis of plant BCAAs has been conducted. The motivations driving the advancement in this area include: 1) human and other animals cannot synthesize these essential amino acids, and have to obtain them directly or indirectly from plants [1], [2]; 2) enzymes in their biosynthetic pathways are targets of several economically important herbicides [3].

In plants, Val and Ile are synthesized in two parallel pathways using a single set of enzymes catalyzing the reactions with different substrates. The pathway toward Leu synthesis branches off from 2-oxoisovalerate, an intermediate of Val biosynthesis [3]. The reaction follows a three-step pathway to generate a new 2-oxoacid, which is elongated by a methylene group. Similar 2-oxoacid-based chain-elongation reactions are also used in other biosynthetic pathways, such as the tricarboxylic acid (TCA) cycle, lysine (Lys) biosynthesis in fungi and methionine (Met) chain-elongation in the biosynthesis of aliphatic glucosinolates found in *Brassicaceae* plants including *Arabidopsis thaliana* [1]. The notion that Met chain-elongation cycle is evolutionarily recruited from Leu biosynthetic pathway has been well supported by several independent experiments [4]–[8].

Although some pathway connections and crosstalk have been documented in plants, the relationship between Leu metabolism and other cellular metabolic pathways remains largely unclear. In the course of our characterization of the functions of Arabidopsis isopropylmalate dehydrogenases (*IPMDHs*) in Leu and glucosinolate biosynthesis, we observed that the phenotypic aberrations caused by genetic disruption of *IPMDH2* and *IPMDH3* can be further exaggerated by the simultaneous loss of *IPMDH1*. Likewise, the glucosinolate profile changes in the *ipmdh1* mutant can be enhanced by concurrent loss of *IPMDH2* and *IPMDH3* alleles [6], [7]. To explore the effect of such gene-dosage dependent alterations on cellular metabolic changes, we have carried out quantitative proteomics and metabolomics of the *IPMDH* mutants in order to determine metabolic pathways affected by the perturbation of Leu and glucosinolate homeostasis. Our results have revealed that the absence/decrease of IPMDHs, key enzymes in the biosynthesis of Leu and glucosinolates, can trigger a wide spectrum of metabolic changes in a gene-dosage dependent manner, including imbalance of amino acid homeostasis, changes in aliphatic and indolic glucosinolate biosynthesis, decrease of photosynthesis, enhancement of protein synthesis, alteration of redox status. These results constitute important information toward understanding plant molecular networks involving Leu primary metabolism and glucosinolate specialized metabolism.

## Materials and Methods

### Plant growth, Genotyping and RT-PCR

Seeds of *Arabidopsis thaliana* (L.) Heynh ecotype Columbia (Col-0) (Wild-type), and Salk mutant lines *ipmdh1* (Salk_063423), *ipmdh2* (Salk_152647) and *ipmdh3* (Salk_013237) were obtained from the Arabidopsis Biological Resource Center (ABRC; Columbus, OH, USA). Mutants of *IPMDH1/IPMDH1 IPMDH2/ipmdh2 ipmdh3/ipmdh3* (*AABbdd*) and *ipmdh1/ipmdh1 IPMDH2/ipmdh2 ipmdh3/ipmdh3* (*aaBbdd*) were created as previously described [8]. Seeds were surface sterilized and germinated on Murashige and Skoog medium in a controlled growth chamber under a 16 h light/8 h dark cycle with 23°C day/20°C night. After 10 days, plant seedlings were transferred to moistened soil and grown under the same conditions as previously described [6]. The genotypes of the single, double and triple mutants were published [6], [8]. Total RNA isolation and RT-PCR were conducted as previously described [6]. Primers used for RT-PCR were shown in Supporting Information Table S1.

### Protein Extraction and Isobaric Tag for Relative and Absolute Quantification (iTRAQ)

Leaf proteins from wild-type and the three mutant lines were extracted according to a previous method [9]. Protein concentration was determined using an EZQ assay kit (Invitrogen Inc., USA). A total of 100 µg protein of each sample was solubilized, reduced and alkylated, and trypsinized, followed by labeling using 8-plex iTRAQ kits according to manufacturer instructions (AB Sciex Inc., USA). The samples of wild-type were labeled with iTRAQ tags 113 and 117, single mutants (*aaBBDD*) with tags 114 and 118, double mutants (*AABbdd*) with tags 115 and 119, and triple mutants (*aaBbdd*) with tags 116 and 121. Two independent iTRAQ experiments were carried out, thus each sample has four biological replicates. After one hour incubation at room temperature, the labeling reaction was quenched by adding 100 µl distilled water. The labeled samples were pooled and lyophilized. Each set of the labeled samples was dissolved in 1 ml loading buffer (15 mM KH_2_PO_4_ in 25% acetonitrile, pH 3.0) for strong cation exchange (SCX) fractionation as previously described [10].

Each SCX fraction was submitted to a Triple TOF 5600 system coupled with an Ultra 2D Plus nanoflow HPLC (Eksigent Inc., USA). Online trapping, desalting, and separation were conducted as previously described [10]. Protein identification and relative quantification were performed with a ProteinPilot™ software 4.0 (AB Sciex Inc., USA) using an Arabidopsis database with 62501 entries (downloaded on March 12, 2010) [10]. A 1.3-fold cut-off was chosen to determine increased and decreased proteins in addition to a p-value of less than 0.05 in at least two biological replicates.

### Two-dimensional Difference Gel Electrophoresis (2D-DIGE) and Protein Identification

Protein samples, each with three biological replicates, were labeled with CyDyes (Cy2, Cy3, or Cy5) according to the three dye protocol for minimal labeling (GE Healthcare Inc., USA). The design of labeling sets was shown in Supporting Information Table S2. Briefly, protein pellets were reconstituted in 30 mM Tris-HCl, pH 8.5, containing 7 M urea, 2 M thiourea, and 4% (w/v) CHAPS with vortex mixing for 30 min at 25°C followed by centrifugation for 15 min at 14,000 g to remove insoluble material. Then, 50 mg of protein were adjusted to final volume of 10 µL. One microliter of dye (100 pmol) was added and the mixture was incubated on ice in the dark for 30 min. The labeling reaction was stopped by adding 1 µL 10 mM lysine and incubation on ice for 10 min. Dye swapping between experimental samples was performed to control dye-specific artifacts resulting from preferential labeling or variable fluorescence characteristics of the CyDyes. After labeling, the samples were mixed and loaded onto 24-cm ReadyStrip IPG Strips (pH 4–7 nonlinear) (Bio-Rad Inc., USA). Isoelectric focusing (IEF), SDS gel electrophoresis, and imaging were conducted as previously described [11]. The gel images were analyzed using Decyder software (GE Healthcare, USA). Differentially expressed protein spots with statistical significance (p<0.05) were excised using a ProPic robotic picker (Genomic Solutions, USA). Protein in-gel digestion and identification using mass spectrometry (MS) were conducted as previously described [12], except the database used was the Arabidopsis database.

### Metabolite Extraction and Metabolomic Analysis

Metabolites were extracted from 250 mg 4-week-old rosette leaves of wild-type and the *IPMDH* mutants. Four biological replicates were analyzed for each sample. Metabolomic analysis was conducted using ultra high performance liquid chromatography (UPLC)-MS/MS and gas chromatography (GC)-MS. Detailed experimental procedures can be found in previous publications [13]–[15].

Statistical analyses including ANOVA, Tukey's test, Welch's t-test and/or Wilcoxon's rank sum test were performed using SAS Enterprise software (Version 4.3) and the R package (http:///www.r-project.org). The identified proteins and metabolites were clustered according to the log_2_ values of the ratios of their levels in the mutants over in the wild-type control.

## Results

### Gene-dosage Dependent Perturbation of Arabidopsis *IPMDH*s

**Figure 1:**
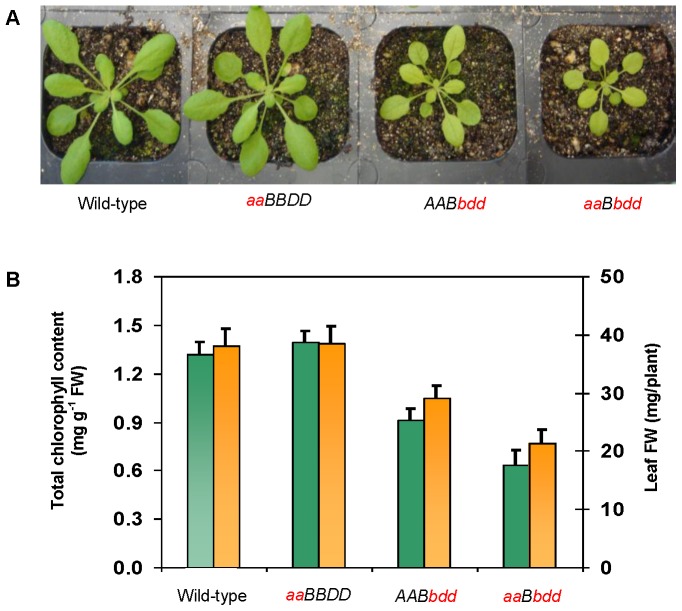
Morphological phenotype, total chlorophyll contents, and fresh weight of the Arabidopsis isopropylmalate dehydrogenase (*IPMDH*) mutants. (A) Phenotypes of the four-week-old *IPMDH* mutants. (B) Chlorophyll contents (green columns) and leaf fresh weight (orange columns) of the *IPMDH* mutants. The bars showed standard errors of seven different plants. A, *IPMDH1*; a, *ipmdh1*; B, *IPMDH2*; b, *ipmdh2*; D, *IPMDH3*; d, *ipmdh3*; FW, fresh weight. Please note that the phenotype data for the *AABbdd* and *aaBbdd* mutants based on another experiment were published in He et al., 2011a. New Phytologist 189: 160–175.

Single Arabidopsis *IPMDH1* mutant (*ipmdh1/ipmdh1 IPMDH2/IPMDH2 IPMDH3/IPMDH3*, *aaBBDD*), double *IPMDH2* (heterozygous) *IPMDH3* mutant (*IPMDH1/IPMDH1 IPMDH2/ipmdh2 ipmdh3/ipmdh3*, *AABbdd*), and triple *IPMDH* mutant (*ipmdh1/ipmdh1 IPMDH2/ipmdh2 ipmdh3/ipmdh3*, *aaBbdd*) were established, and their morphological and physiological characteristics were characterized in our previous studies [6], [8]. Four-week-old *ipmdh1* mutant did not exhibit obvious morphological phenotypes (Figure 1A). In contrast, the double and triple mutants showed reduced leaf size, fresh weight, and chlorophyll contents (Figures 1A and 1B). In addition, glucosinolate profiles were significantly altered in the single and triple mutants [6], [8]. More importantly, the morphological phenotype and glucosinolate chemotype found in the single or double mutants were further enhanced in the triple mutant, indicating that the IPMDHs function in Leu and glucosinolate metabolism in a gene-dosage dependent manner. Through investigating the changes of proteotypes and chemotypes in these mutants, we aim to not only better understand the phenotypic changes, but also reveal the intricate molecular networks underlying cellular acclimation to the genetic perturbation of Leu and glucosinolate biosynthesis.

### Differentially Expressed Proteins in the *IPMDH* Mutants Identified Using iTRAQ

**Figure 2:**
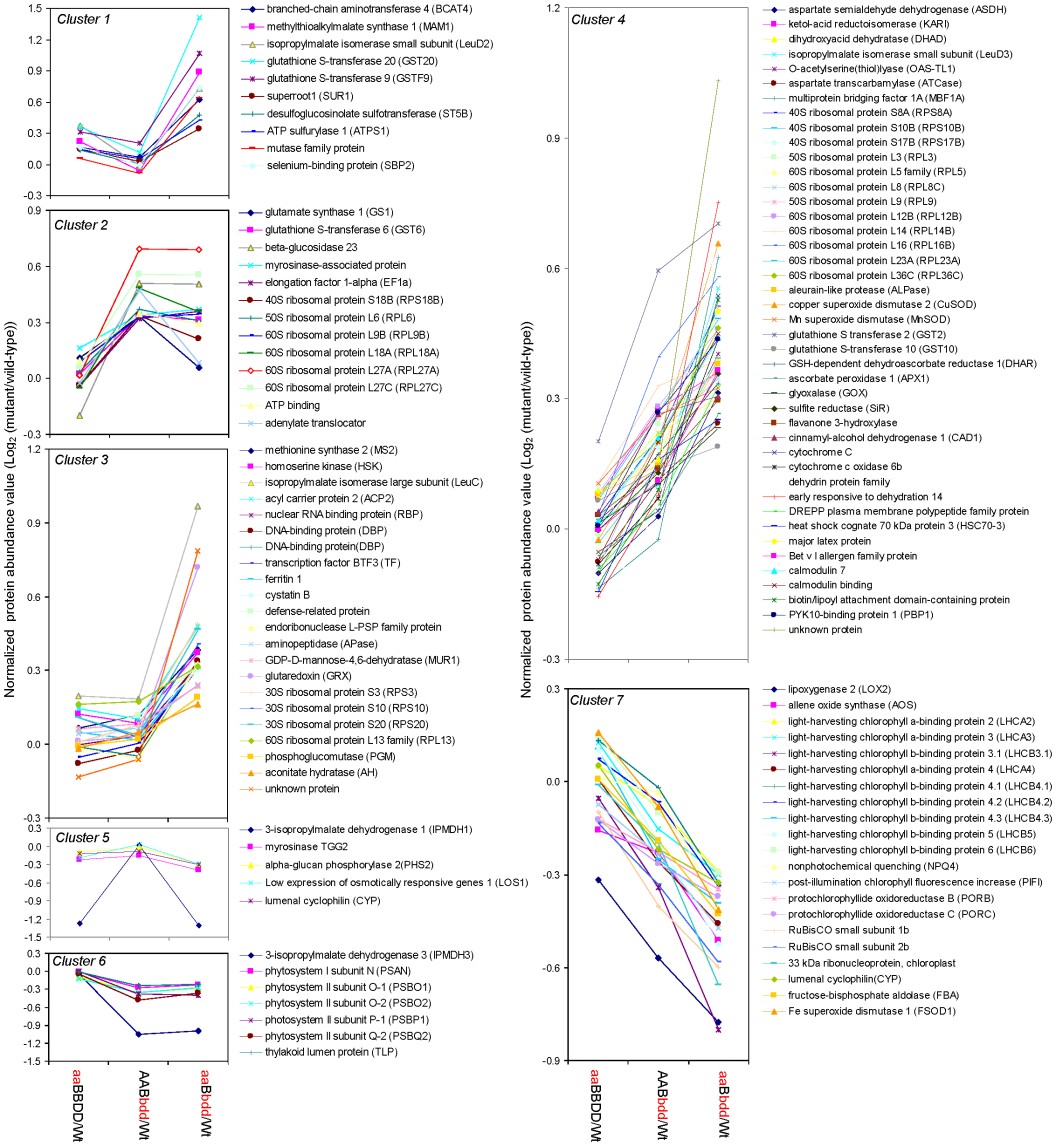
Clusters of differentially expressed proteins in the *IPMDH* mutants identified by iTRAQ. Plotted are the log2 ratios of the protein levels in the mutants over in wild-type. Cluster 1 includes proteins induced in the triple mutant, but reduced in the double mutant; Cluster 2 has proteins with significant increase in abundance in the double mutant, but the increase is not further enhanced in the triple mutant; Cluster 3 includes proteins with significant increase in levels only in the triple mutant. Cluster 4 shows proteins induced in the double mutant and further enhanced in the triple mutant; Cluster 5 shows proteins with the trend of decrease in the single and triple mutants; Cluster 6 includes proteins with decreased levels in the double and triple mutants; Cluster 7 shows progressive protein decreases in the double and triple mutants. A, *IPMDH1*; a, *ipmdh1*; B, *IPMDH2*; b, *ipmdh2*; D, *IPMDH3*; d, *ipmdh3*.

Two independent iTRAQ experiments were conducted to determine differentially expressed proteins in the three *IPMDH* mutants and wild-type plants, each sample with four biological replicates. Based on the criteria of more than 1.3 fold protein abundance changes between the mutants and the wild-type and *p*-values smaller than 0.05 in at least two replicates, 88 and 33 proteins were identified to be increased and decreased, respectively. To analyze coordinately regulated proteins, the differentially expressed proteins were grouped in seven clusters (Figure 2, Table S3).

The increased proteins were grouped into four clusters: (**1**) Cluster 1 included 10 proteins related to glucosinolate biosynthesis, with increased levels in the single and triple mutants, but not significantly in the double mutant compared to the wild-type. Five of them were well-known to be involved in glucosinolate biosynthesis, including branched-chain aminotransferase 4 (BCAT4) [4], methylthioalkylmalate synthase 1 (MAM1) [16], [17], isopropylmalate isomerase small subunit (LeuD2) [7], [18], superroot1 (SUR1) [19], and desulfoglucosinolate sulfotransferase (ST5B) [20]. Besides, two members of glutathione S-transferase (GST) family (GST20 and GST9) were grouped to this cluster. GSTs or GST-like enzymes were proposed to catalyze the incorporation of reduced sulfur into the glucosinolate core structure [21]. In addition, ATP sulfurylase 1 (ATPS) was also found in cluster 1. ATPS was primarily a sulfur metabolism-related enzyme, and was recently suggested to be part of the glucosinolate metabolic network [22]. The last two proteins in this group, a mutase family protein (AT1G21440) and a selenium binding protein 2 (SBP2), have not been reported to play a role in glucosinolate biosynthesis. Through in silico gene coexpression analysis [23], we found that this mutase gene was tightly co-regulated with a number of glucosinolate biosynthetic genes (Figure S1), suggesting its potential involvement in glucosinolate biosynthesis. In addition, recent studies have shown that the selenium regulates the expression of glucosinolate biosynthetic genes and could affect the glucosinolate metabolism [24], [25]. (**2**) Cluster 2 contained 13 proteins, whose abundances were significantly increased in the double mutant, but not further increased in the triple mutant when wild-type levels were used as denominators. Among them, one elongation factor and six ribosomal proteins were involved in protein synthesis, suggesting protein synthesis was enhanced when *IPMDH2* and *IPMDH3* were mutated. Myrosinase-associated protein is known to be involved in glucosinolate hydrolysis [26]. The other five proteins included GST6, beta-glycosidase 23, glutamate synthase 1(GS1), ATP-binding protein and adenylate translocator. How they are related to *IPMDH* functions is not known. (**3**) Cluster 3 contained 22 proteins, showing strong increase of protein levels in the triple mutant only. That is, these proteins were tremendously induced by the loss of most *IPMDH* alleles. Five transcription-related proteins (two DNA-binding proteins, a RNA binding protein, a transcription factor BTF3, and an endoribonuclease L-PSP family protein), six protein metabolism-related proteins (four ribosomal proteins, an aminopeptidase, and a cystatin B), an acyl carrier protein 2 (ACP2) for fatty acid biosynthesis, and a ferritin for storage of irons were found in this cluster. In addition, it is interestingly to find methionine synthase 2 (MS2), homoserine kinase (HSK), and isopropylmalate isomerase large subunit (LeuC) in this cluster, suggesting that aspartate (Asp)-derived amino acid biosynthesis and the isomerization step in Leu biosynthesis (the step prior to IPMDH catalysis) were induced in the triple mutant. Furthermore, three sugar metabolism-related enzymes (phosphoglucomutase, aconitate hydratase, and GDP-D-mannose-4,6-dehydratase) and two stress and defense-related proteins (a glutaredoxin (GRX) and a defense-related protein) were obviously induced in the triple mutant. (**4**) Cluster 4 represented the largest protein group of 43 proteins. They exhibited continuous expression increase in the double and triple mutants and were involved in amino acid metabolism, protein synthesis and turnover, reactive oxygen species (ROS) scavenging and stress response, fatty biosynthesis, and signaling. Four of them are the enzymes in branched-chain amino acid biosynthesis. They were aspartate semialdehyde dehydrogenase (ASDH), ketol-acid reductoisomerase (KARI), dihydroxyacid dehydratase (DHAD) and isopropylmalate isomerase small subunit (LeuD3), which were coordinately activated in the double and triple mutants. Besides, a transcription factor (multiprotein bridging factor 1A), twelve ribosomal proteins, and an aleurain-like protease were grouped to this cluster, suggesting protein synthesis and turnover were induced in the mutants. In addition, 18 stress/defense-related proteins showed gradual increases in the double and triple mutants. They included seven ROS scavenging enzymes (copper superoxide dismutase 2 (CuSOD), manganese superoxide dismutase (MnSOD), GST2, GST10, glutathione (GSH)-dependent dehydroascorbate reductase 1(DHAR), ascorbate peroxidase 1 (APX1), and glyoxalase (GOX)), five redox-related proteins (sulfite reductase (SiR), flavanone 3-hydroxylase (iron and ascorbate as cofactors), cinnamyl-alcohol dehydrogenase 1 (CAD1), cytochrome C, and cytochrome c oxidase 6b), and six defense-related proteins (dehydrin protein family, early responsive to dehydration 14, DREPP plasma membrane polypeptide family protein, heat shock cognate 70 kDa protein 3 (HSC70-3), major latex protein, and Bet v I allergen family protein). The increased expression of these proteins suggests significant cellular oxidative stress attributed to mutation of the *IPMDH* genes.

Clusters 5 to 7 contained thirty-three decreased proteins in the mutants compared to the wild-type. (**5**) Cluster 5 contained five proteins, which showed reduced expression in the triple mutant. As expected, IPMDH1 was found in this group in respect to its loss-of-function in the single and triple mutants, but not in the double mutant. This finding demonstrates the reliability and robustness of the iTRAQ approach in determining differential proteins. However, the MS result showed that the IPMDH1 protein was massively reduced, but not abolished. To test the possibility of partial gene expression considering the T-DNA insertion at close to the 5′ end (Figure S2A), reverse-transcription PCR (RT-PCR) was performed using the primers designed to amplify the fragment upstream of the T-DNA site. No transcript was observed in *ipmdh1*, confirming that this is a knockout mutant (Figure S2B). Alternatively, we speculate that the residual protein signal in the mutant could be attributed to the precursor cross-contamination recently discovered with the iTRAQ technology [27], [28]. Reduction of IPMDH1 abundance to extremely low levels after background correction supported such a hypothesis (Figure S3). To obtain additional evidence, we conducted independent experiments, where proteins extracted from the *ipmdh1* mutant and wild-type plants were processed using the iTRAQ workflow except without labeling and sample combination. After analyzing the samples separately on the MS using the same conditions, we were able to detect IPMDH1 peptides with high confidence in the wild-type samples, but not in *ipmdh1* mutant (Figure S3). These results provided another line of evidence supporting that the low IPMDH1 peptide signals in the mutants were due to iTRAQ precursor contamination. In addition, one of two main myrosinases in *Arabidopsis* shoot (myrosinase 2) was found to be reduced in the triple mutant, suggesting potential repression of glucosinolate degradation. (**6**) Cluster 6 had seven proteins with similar reduced levels in the double and triple mutants. As expected, IPMDH3 was found to be reduced in this group. In contrast to the *ipmdh1* knockout mutant, we did detect partial transcripts of *IPMDH3* in the *ipmdh3* mutants, although at extremely low levels. Consistent with our previous study [8], no *IPMDH3* transcripts could be detected when using the PCR primers covering the T-DNA insertion site (Figure S2B). The low level truncated transcripts could be responsible for the residual protein level detected in the *ipmdh3* mutants. The other six proteins in this cluster belonged to photosynthesis-related proteins, suggesting that the photosynthetic processes were disrupted by the reduction of the *IPMDHs* in Leu biosynthesis. (**7**) Cluster 7 contained 21 proteins, representing the largest cluster of proteins exhibiting progressive decrease due to the gene-dosage dependent disruption of *IPMDHs*. To our surprise, lipoxygenase 2 (LOX2) and allene oxide synthase (AOS), two rate-limiting enzymes involved in jasmonate (JA) biosynthesis, were reduced due to reduction of IPMDH2 and IPMDH3 primarily in Leu biosynthesis [29]. Such a potential connection has not been reported. In addition, 17 out of 21 proteins in this cluster belonged to photosynthesis-related proteins (Figure 2), suggesting progressive deterioration of photosynthesis, as displayed by the phenotype of chlorosis and reduced growth in the double and triple mutants (Figure 1).

### Differentially Expressed Proteins in the *IPMDH* Mutants Identified Using 2D-DIGE

**Figure 3:**
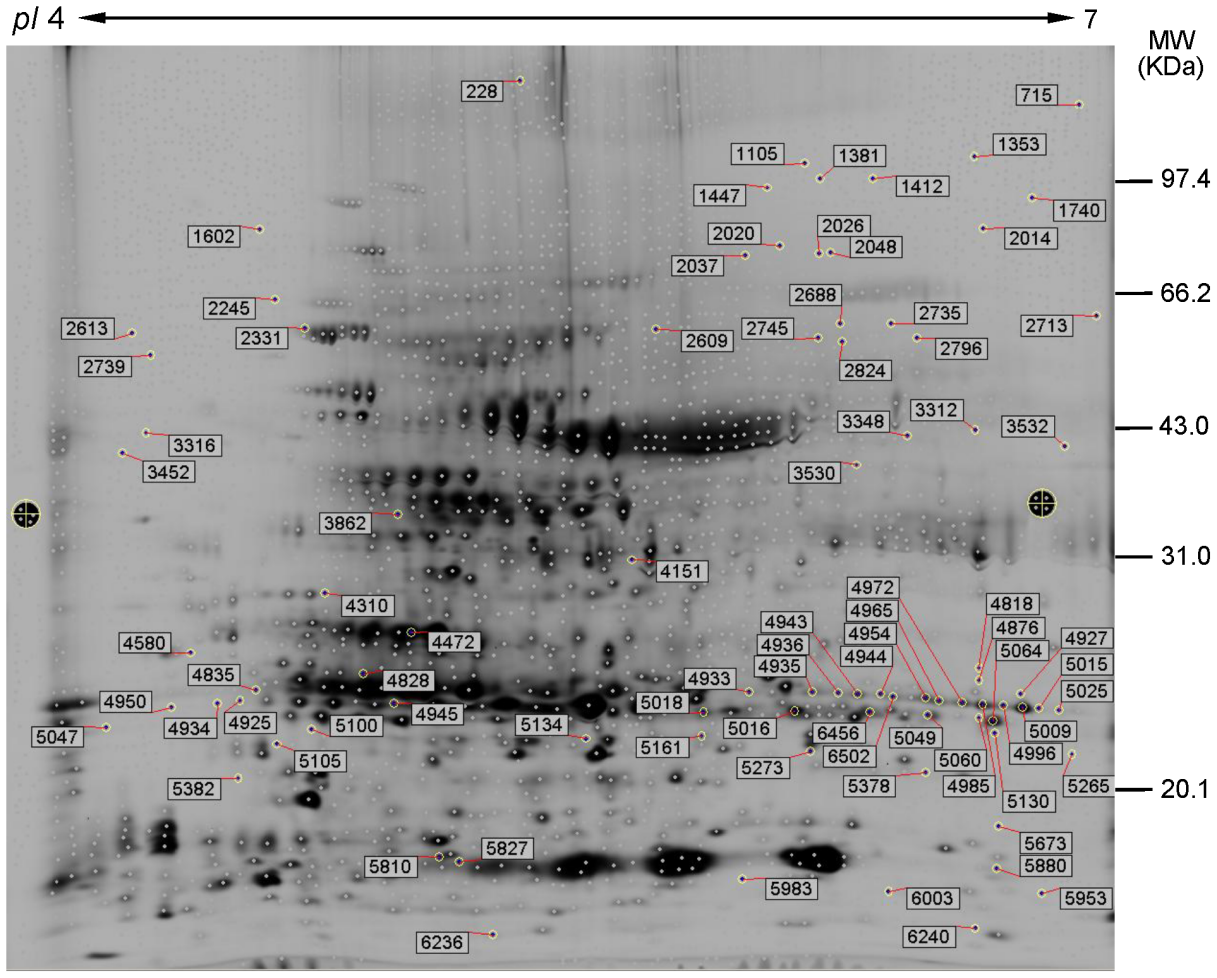
Representative 2D-DIGE map of differentially expressed proteins in the *IPMDH* mutants and wild-type plants. The proteins samples from the mutants of *IPMDH1*, *IMPDH2*, and *IPMDH3*, and wild-type were labeled with Cy2, Cy3, and Cy5 and then separated on 24 cm IPG strips (pH 4–7 linear gradient) through isoelectric focusing (IEF) in the first dimension, followed by 12.5% SDS-PAGE gels in the second dimension. Molecular weight (MW) in kilodaltons and pI of proteins are indicated on the right and top of gel, respectively. A total of 84 differentially expressed proteins marked with spot numbers were identified by MS/MS. For detailed information, please refer to Table S2.

Complementary to iTRAQ analysis, 2D-DIGE of the same protein samples identified 84 proteins, with 25 increased and 26 decreased proteins in the mutants (Table S2). Among them, 10 proteins have been identified in the iTRAQ analysis. In Figure 3, a representative 2D-DIGE gel was shown with the differentially expressed protein spots identified by MS. In addition, several proteins were represented by multiple spots on the gel (Table S2), suggesting potential posttranslational modifications of the proteins.

**Figure 4:**
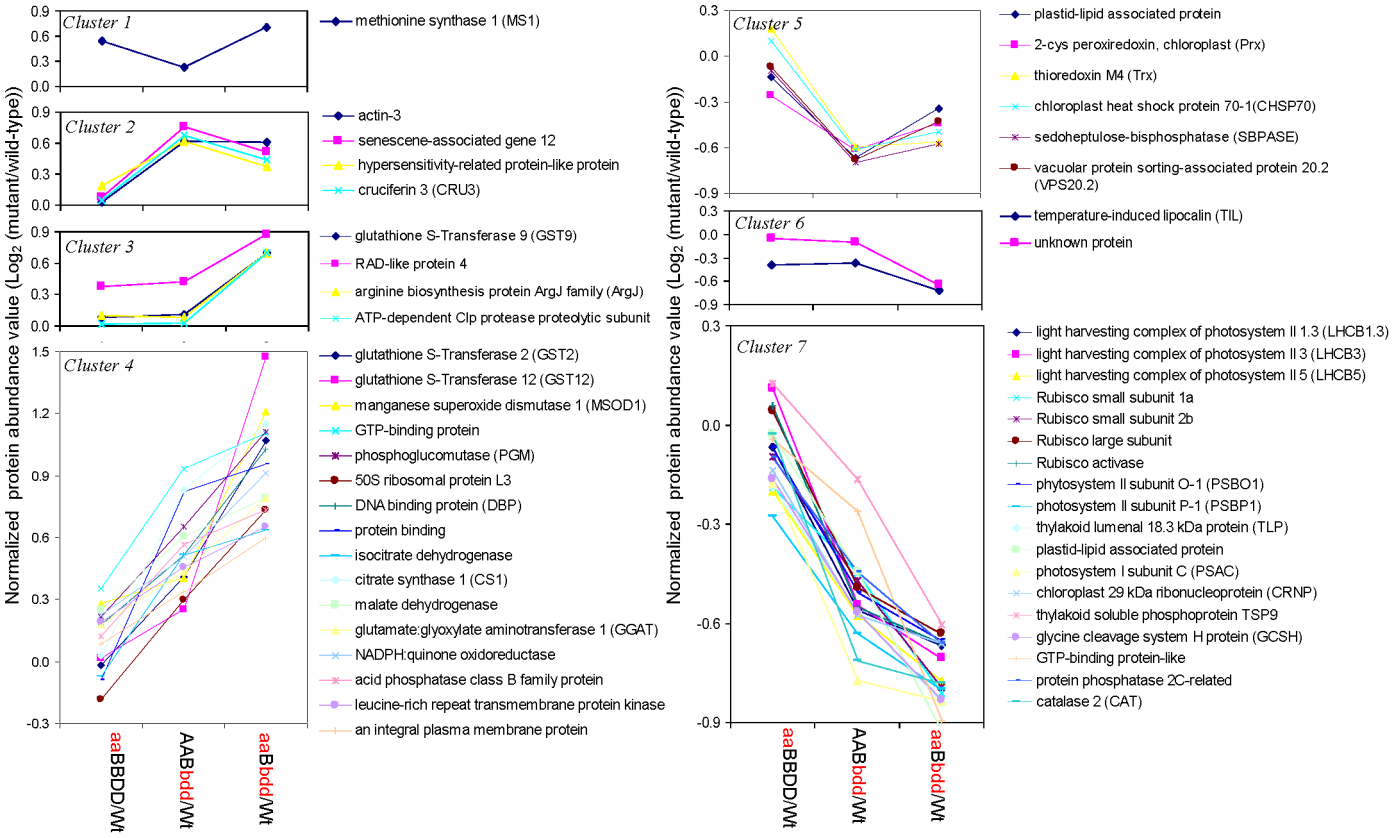
Clustering of proteins identified by 2D-DIGE with similar trends of differential expression in the *IPMDH* mutants. Plotted are the log2 ratios of the protein levels in the mutants over in wild-type. Cluster 1 includes proteins induced in the triple mutant, but reduced in the double mutant; Cluster 2 has proteins with significant increase in abundance in the double mutant, but the increase is not further enhanced in the triple mutant; Cluster 3 includes proteins with significant increase in levels only in the triple mutant. Cluster 4 shows proteins increased in the double mutant and further enhanced in the triple mutant; Cluster 5 shows proteins with the trend of decrease in the single and triple mutants; Cluster 6 includes proteins with decreased levels in the double and triple mutants; Cluster 7 shows progressive protein decreases in the double and triple mutants. A, *IPMDH1*; a, *ipmdh1*; B, *IPMDH2*; b, *ipmdh2*; D, *IPMDH3*; d, *ipmdh3*.

Based on the clustering method used for the iTRAQ data, the induced proteins in the mutants were grouped into four clusters (Figure 4). (**1**) Methionine synthase 1 (MS1) in cluster 1 was strongly increased in single and triple mutants, suggesting that Met synthesis was highly responsive to the disruption of the chain-elongation cycle in glucosinolate biosynthesis. (**2**) Actin 3, two stress-related proteins (senescence-associated gene 12 and hypersensitivity-related protein), and cruciferin 3 (CRU3) were grouped into this cluster, with increased levels in the double and triple mutants. (**3**) GST9, Ras-related (RAD)-like protein 4, arginine biosynthesis protein ArgJ (ArgJ), and ATP-dependent Clp protease subunit were induced only in the triple mutant. (**4**) Cluster 4 included 16 proteins that showed progressive increases in abundance in the double and triple mutants. Four of them were glycolysis and TCA cycle-related enzymes, including phosphoglucomutase (PGM), isocitrate dehydrogenase (IDH), citrate synthase 1 (CS1), and malate dehydrogenase (MDH). Four other proteins were redox-related enzymes, i.e., GST2, GST12, MnSOD1, and NADPH quinone oxidoreductase. In addition, three signaling and membrane-related proteins (GTP-binding protein, Leu-rich repeat transmembrane protein kinase, and integral plasma membrane protein), as well as four proteins involved in transcription and proteins/amino acid metabolism (DNA binding protein, protein binding, 50S ribosomal protein L3, and glutamate: glyoxylate aminotransferase 1 (GGAT)) were identified in this cluster.

The 26 decreased proteins in the mutants were grouped into three clusters. (**5**) Six proteins exhibited decreased levels in the double mutant, but with no further reduction in the triple mutant (Figure 4). They were a Calvin cycle enzyme (sedoheptulose-bisphosphatase (SBP)), a chloroplast heat shock protein 70-1 (CHSP70), and four redox-related proteins including 2-cys peroxiredoxin (Prx), thioredoxin M4 (Trx), plastid-lipid associated protein (fibrillin). (**6**) Cluster 6 included chlororespiratory reduction 41 (CRR41) and temperature-induced lipocalin (TIL) that decreased in the triple mutant, but not in the single and double mutants. (**7**) Cluster 7 contained 18 proteins that showed gradual decreases in abundance in the double and triple mutants. Among them, 15 were photosynthesis-related proteins, i.e., four light harvesting complex (LHC) subunits of photosystem II (PSII), photosystem I subunit C (PSI C), two Rubisco small subunits, a Rubisco large subunit, a Rubisco activase, two thylakoid lumenal proteins (TLPs), a chloroplast 29 kDa ribonucleoprotein (CRNP), a fibrillin, and a glycine cleavage system H protein (GCSH). These results are consistent with the iTRAQ result showing disruption of photosynthesis in the mutants.

### Metabolomic Changes in the *IPMDH* Mutants

**Figure 5:**
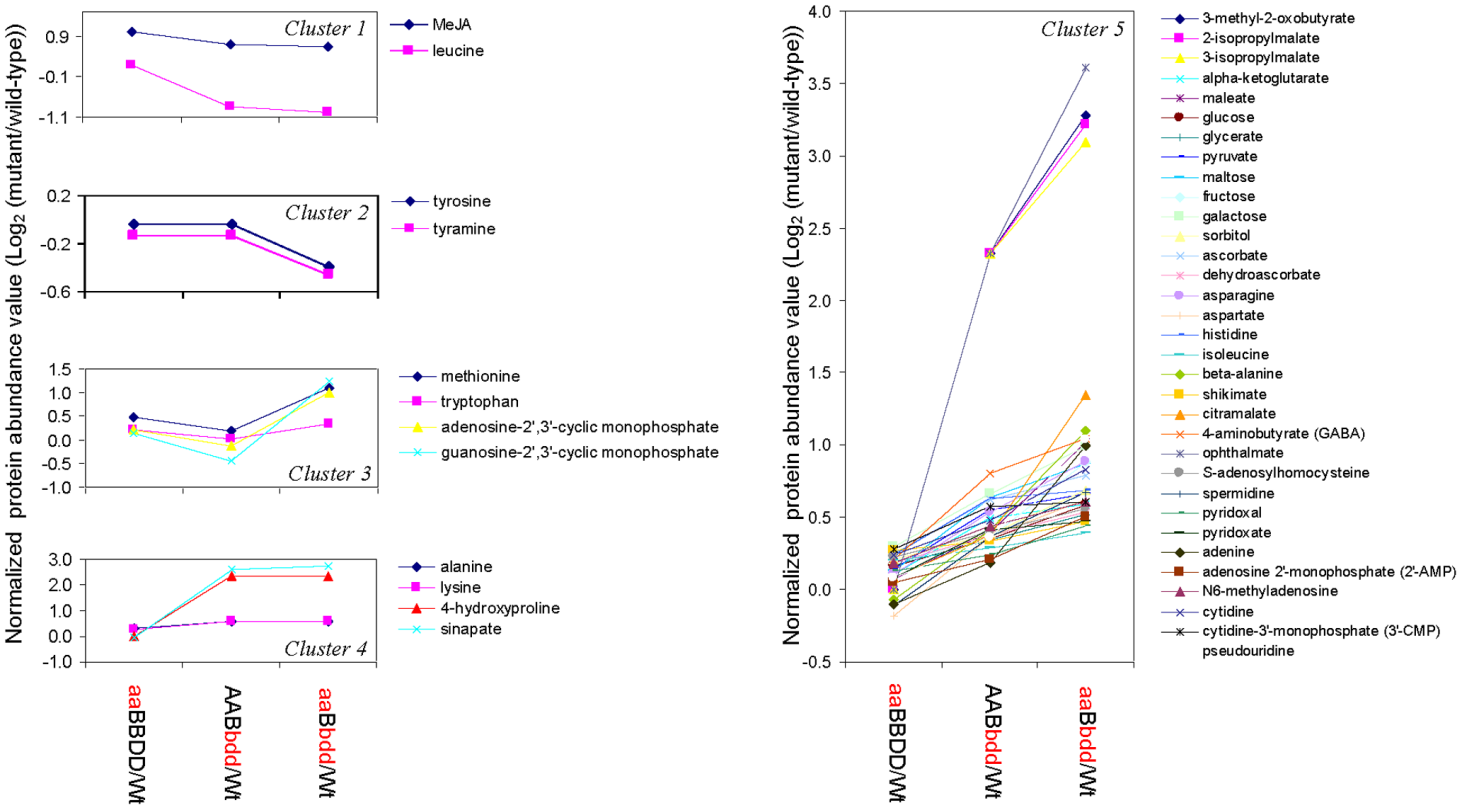
Clusters of metabolites with similar trends of changes in the *IPMDH* mutants. Plotted are the log2 ratios of the metabolite levels in the mutants over in wild-type. Cluster 1 shows metabolites decreased in levels in either the double or triple mutant; Cluster 2 includes metabolites decreased only in the triple mutant; Cluster 3 shows metabolites reduced in the double mutant, but incresed in the triple mutant; Cluster 4 includes metabolites increased in the double mutant, but the increase was not further enhanced in the triple mutant; Cluster 5 shows metabolites induced in the double mutant and further enhanced in the triple mutant. A, *IPMDH1*; a, *ipmdh1*; B, *IPMDH2*; b, *ipmdh2*; D, *IPMDH3*; d, *ipmdh3*.

To determine metabolite alterations in the mutants, the levels of 64 metabolites, mostly primary metabolites were analyzed using GC-MS and UPLC-MS. A total of 45 metabolites exhibited significant changes and were grouped into five clusters based on the trend of changes (Figure 5). Cluster 1 had Leu and methyl jasmonate (MeJA) that showed decreased levels in the double and triple mutants. The Leu reduction is expected in the *IPMDH* mutants [8]. The MeJA decrease correlates well with the decreases of LOX2 and AOS found in the proteomic analysis (Figure 2). Cluster 2 included tyrosine (Tyr) and tyramine that were reduced only in the triple mutant. Met and tryptophan (Trp) in cluster 3 were induced in the single and triple mutants. In cluster 4, the levels of alanine (Ala), lysine (Lys), 4-hydroxyproline, and sinapate increased in the double and triple mutants. Cluster 5 contained more than 75% of the examined metabolites with increases in the gene-dosage dependent manner in the mutants (Figure 5, Table S4). It is interesting to note that Met, the precursor of aliphatic glucosinolate biosynthesis was found to be slightly increased in the single mutant, and considerably enhanced in the triple mutant. This was consistent with the proteomics results that Met biosynthesis enzymes (MS1 and MS2) were induced (Figures 2 and 4). Importantly, three intermediates upstream of the oxidative decarboxylation step in Leu biosynthesis (i.e., ketoisovalerate, 2-isopropylmalate (2-IPM), and 3-isopropylmalate (3-IPM)) were substantially accumulated in the double and triple mutants, but not detectable in the wild-type plants, supporting the direct participation of IPMDH2 and IPMDH3 in Leu biosynthesis (Figure S4). Moreover, several metabolites in carbohydrate metabolism (e.g., glucose, fructose, sorbitol, maltose, galactose, ascorbate, and glycerates) and TCA cycle (pyruvate and 2-oxo-glutarate) were substantially accumulated in the gene-dosage dependent manner (Figure 5). Interestingly, five amino acids were increased in the double and triple mutants, such as β-Ala, asparagine (Asn), Asp, histidine (His), and Ile (Figure 5). In addition, the mutants had higher levels of sinapate, suggesting induction of the phenylpropanoid pathway, a response indicative of cellular stress. Last but not least, ascorbate and ophthalmate showed progressive increase in the double and triple mutants, implying induction of the glutathione pathway in the mutants.

## Discussion

### Gene-dosage Dependent Perturbation of Amino acid Homeostasis in the *IPMDH* Mutants

**Figure 6:**
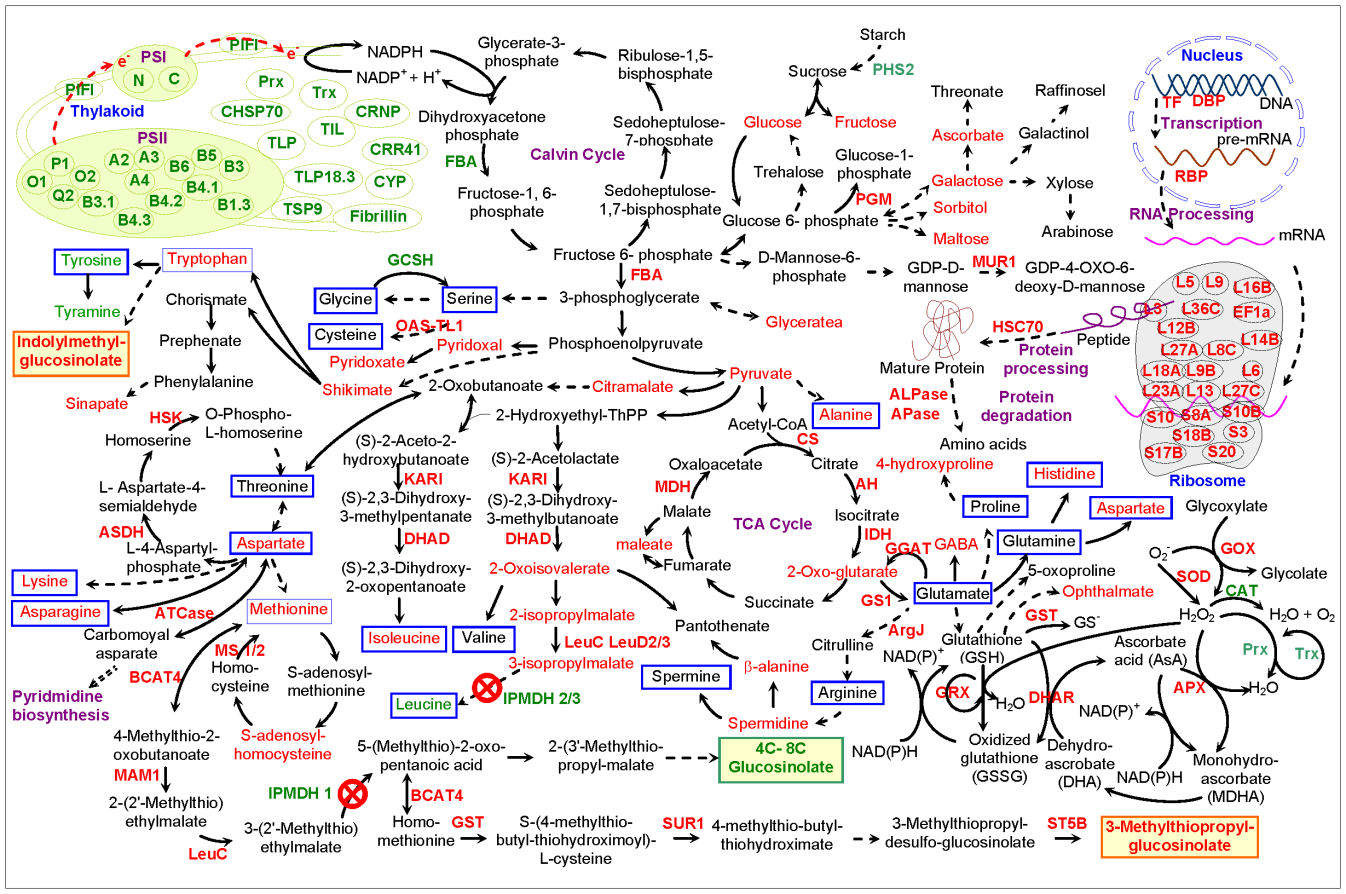
Schematic presentation of proteins and metabolites in the *IPMDH* mutants. Proteins and metabolites in red indicate increased and in green decreased in the mutants compared to wild-type. AH, aconitate hydratase; ALPase, aleurain-like protease; APase, aminopeptidase; APX, ascorbate peroxidase; ArgJ, arginine biosynthesis protein ArgJ; AsA, ascorbate acid; ASDH, aspartate semialdehyde dehydrogenase; ATCase, aspartate transcarbamylase; BCAT, branched-chain aminotransferase; CAT, catalase; CHSP70, chloroplast heat shock protein 70; CRNP, chloroplast 29 kDa ribonucleoprotein; CRR, chlororespiratory reduction; CS, citrate synthase; CYP, luminal cyclophilin; DBP, DNA-binding protein; DHA, dehydroascorbate; DHAD, dihydroxyacid dehydratase; DHAR, dehydroascorbate reductase; FBA, fructose-bisphosphate aldolase; GABA, γ-aminobutyrate; GCSH, glycine cleavage system H protein; GGAT, glutamate:glyoxylate aminotransferase; GOX, glyoxalase; GRX, glutaredoxin; GS, glutamate synthase; GSH, glutathione; GSSG, oxidized glutathione; GST, glutathione S transferase; HSC, heat shock cognate; HSK, homoserine kinase; IDH, isocitrate dehydrogenase; IPMDH, 3-isopropylmalate dehydrogenase; KARI, ketol-acid reductoisomerase; LeuC, isopropylmalate isomerase large subunit; LeuD, isopropylmalate isomerase small subunit; MAM, methylthioalkylmalate synthase; MDH, malate dehydrogenase; MDHA, monohydroascorbate; MUR, GDP-D-mannose-4,6-dehydratase; MS, methionine synthase; OAS-TL, O-acetylserine (thiol) lyase; PGM, phosphoglucomutase; PHS, alpha-glucan phosphorylase; PIFI, post-illumination chlorophyll fluorescence increase; Prx, 2-cys peroxiredoxin; PSI, photosystem I; PSII, photosystem II; RBP, RNA binding protein; SOD, superoxide dismutase; ST5B, desulfoglucosinolate sulfotransferase; SUR, superroot; TCA, tricarboxylic acid; TF, transcription factor; TIL, temperature-induced lipocalin; TLP, thylakoid lumenal protein; Trx, thioredoxin; TSP, thylakoid soluble phosphoprotein.

Amino acid homeostasis is essential for plant growth, development and defense [30]. Plant amino acid homeostasis is regulated by *de novo* biosynthesis, uptake/translocation, and protein synthesis/turnover. End product inhibition at the branching points of the biosynthesis of BCAAs (i.e. Leu, Ile and Val) is pivotal to balance the fluxes between different amino acid pathways. On one hand, external environmental stresses (e.g., pathogen infection) or supply of feedback-competent amino acids could perturb cellular amino acid homeostasis [31]. On the other hand, over-expression of feedback-insensitive orthologs (e.g., anthranilate synthase, acetohydroxyacid synthase, or dihydrodipicolinate synthase) can lead to the increase of Trp, Lys and Val, respectively [32]–[34]. Our previous study discovered that the *imdh2 ipmdh3* mutant was lethal in male gametophytes and had reduced transmission through female gametophytes, probably caused by the decrease of Leu biosynthesis [7]. However, the underlying mechanisms were not known. In this present study, the knockout/knockdown of *IPMDHs* was found to affect amino acid homeostasis in a gene-dosage dependent manner. The Leu contents were gradually decreased in the double mutant (*IPMDH2/ipmdh2 ipmdh3/ipmdh3*) and triple mutant (*ipmdh1/ipmdh1 IPMDH2/ipmdh2 ipmdh3/ipmdh3*). This result supports the direct involvement of IPMDHs in *de novo* Leu biosynthesis, and suggests that other pathways (e.g., protein turnover) are not adequate to sustain the Leu homeostasis in planta. Interestingly, Tyr is the only other amino acid found to be reduced in the mutants, and the cause is not clear (Figure 5). The contents of Met are slightly increased in the single mutant, but significantly induced in the triple mutant. This is consistent with the results from previous studies, i.e., disruption of aliphatic glucosinolate biosynthesis led to increased levels of the Met precursor [4], [6], [35]. For instance, approximately 50% reduction of aliphatic glucosinolates in *bcat4* knockout mutant was concomitant with the increased levels of Met and S-methylmethionine [4]. Another interesting crosstalk between aliphatic and indolic glucosinolates was observed before, i.e., reduced levels of aliphatic glucosinolates are often accompanied with increased levels of indolic glucosinolates [6], [8], [26]. Our metabolomics data showed that the precursor of indolic glucosinolates, Trp, was significantly increased in the single and triple mutants, suggesting such a cross-talk may originate from the *de novo* biosynthesis in addition to share components along the pathways. In addition, the levels of Ala and Lys were elevated in the double and triple mutants to a similar extent, suggesting that the two amino acids are associated with Leu biosynthesis. Furthermore, the contents of Asn, Asp, His and Ile were increased in the double mutant and further enhanced in the triple mutant, indicating that the alteration of these amino acids were associated with the gene-dosage loss of *IPMDHs* in Leu biosynthesis (Figure 5). Consistently, our proteomics results revealed that 11 amino acid metabolism-related enzymes were induced in the *IPMDH* mutants in the gene-dosage dependent manner (Figures 2 and 4). Among them, four enzymes are involved in the biosynthesis Asp-derived amino acid, i.e., ASDH, HSK, KARI and DHAD, consistent with the aforementioned change of the corresponding amino acids. In addition, Three Leu synthesis enzymes, LeuC, LeuD2 and LeuD3, encoding the large subunit and the small subunits of IPMIs, were increased significantly. Moreover, the increased levels of O-acetylserine (thio) lyase 1 (OAS-TL1) and MS2 are consistent with the observed increase of Met (Figure 5). In the correlated amino acid pathways, Lys, Met and threonine (Thr) are synthesized from Asp via different branches of the Asp pathway, and Asp is synthesized from glutamine (Gln) or Asn. Importantly, Thr is the up-stream substrate for the BCAAs, and the synthesis of BCAAs and Gln depends on pyruvate and TCA cycle intermediates (Figure 6). In addition, the three BCAA pathways share common enzymes, such as KARI and DHAD. Our ‘omics’ data have clearly shown the interconnection of the amino acid pathways. For example, the decreased Leu content in the *IPMDH2/3* mutants activated the expression up-stream enzymes (e.g., KARI, DHAD, LeuC, and LeuD2/3). In the meantime, the intermediates upstream of IPMDHs (i.e. 2-oxoisovalerate, 2-IPM, and 3-IPM) in BCAA synthesis accumulated substantially, and promoted the synthesis of Asp, Met, Lys, Asn, Ala, and Tyr, as well as the accumulation of related intermediates (e.g., S-adenosyl-homocysteine, spermidine, β-Ala, citramalate and pyruvate) as well as enzymes (GS1, GGAT and ArgJ) (Figures 2, 4, 5, 6).

The perturbed amino acid homeostasis and the accumulation of specific amino acids in the mutants could alter proteins synthesis and turnover. This was supported by the induction of 16 ribosomal protein large subunits and seven ribosomal protein small subunits in the *IPMDH* mutants. On the other hand, the increases of HSC70, alkaline phosphatase (ALPase), and aminopeptidase (APase) in the mutants suggest enhanced protein processing and degradation in the mutants. The highly dynamic changes of ribosomal proteins in response to different environmental factors were reported before [36]. It is speculated that the changed composition of ribosomal proteins could affect mRNA preference and thus bias the translational efficiency toward a subset of genes crucial for adapting to the environment [37]. These results suggest that the disturbed amino acid homeostasis can lead to the nutrition and proteome imbalance, and ultimately lead to chlorosis and biomass reduction in the *IPMDH* mutants.

### Coordinated Regulation of Glucosinolate Biosynthesis in the *IPMDH* Mutants

It has been observed that genes involved in the same biosynthetic pathway tend to be co-regulated [38]–[40]. This appears to be the case in glucosinolate biosynthesis [23], [41]–[43]. The genes in Met-chain elongation, core structure biosynthesis and secondary modification have been found to exhibit tight co-regulation [23]. Under sulfur-deficiency conditions, the genes involved in glucosinolate biosynthesis were coordinately declined in expression [44], [45]. In this study, the accumulation of Met and Trp provided abundant substrates for glucosinolate biosynthesis in the *IPMDH* mutants. The contents of C3 glucosinolates and indole glucosinolates showed gene-dosage dependent increases in the single and triple mutants, but the contents of C4–C8 glucosinolates were decreased in the single and triple mutants (Figure 1). This result suggests that *IPMDH1* is important for the synthesis of C4 and longer chain glucosinolates, but not for C3 and indole glucosinolate synthesis. This interesting phenomenon has also been observed in the mutants of the early steps of Met-chain elongation [5], [16], [42]. The molecular basis underlying the crosstalk between long-chain aliphatic glucosinolates and C3 or indolic glucosinolates deserves further investigation.

Our proteomics results have shown that nearly all the enzymes involved in glucosinolate synthesis, including MS1, MS2, BCAT4, MAM1, LeuC, GST, SUR1, and ST5B, were coordinately increased in the *IPMDH* mutants (Figures 2, 4 and 6), suggesting that the pathway was coordinately activated when one of the nodes, IPMDH1, was disrupted. Here we propose two complimentary mechanisms to explain the coordination, i.e., end-product feedback induction and intermediate mediated feed-forward induction. In the mutants, C4 glucosinolates were considerably decreased, and the longer chain glucosinolates were close to being completely abolished. It is likely that the reduction or the absence of one or multiple glucosinolates play a regulatory role in controlling the general flux of glucosinolate biosynthesis. Alternatively, the substantial accumulation of the intermediates in the methionine chain-elongation cycle may activate downstream enzymes for accelerating the conversion.

### Cellular Redox Status and Respiration were Affected by the *IPMDH* Mutation

Based on the facts that amino acid synthesis involves nitrate reduction and carbon oxidation, which were reported to associate with NAD redox status and respiratory pathway [46], and induced GSH synthesis could lead to increases in some amino acid levels [47], it is reasonable to hypothesize that perturbation of amino acid homeostasis as described in the previous sections may affect cellular redox status. Here we have found that most of the ROS scavenging enzymes (i.e., SOD, APX, GOX, DHAR, GRX and GST) were induced, while catalase (CAT), Prx, and Trx were reduced in the *IPMDH* mutants (Figures 2, 4 and 6). These results imply that amino acid imbalance affected the cellular redox status in the *IPMDH* mutants. In the cells, excess O_2_^−^ could be converted to H_2_O_2_ and O_2_ in SOD and GOX pathway, and H_2_O_2_ could be eliminated by the ascorbate (AsA)-GSH cycle, but not the CAT and Prx/Trx pathway (Figure 6). In this process, the APX reduces H_2_O_2_ to H_2_O using AsA that undergoes a series of redox reactions by the activities of DHAR and GRX, as well as GST that catalyzes the conjugation of GSH to different hydrophobic and electrophilic compounds (Figure 6).

The changes of GRX and Trx levels are likely to affect the redox status of the enzymes involved in amino acid synthesis and respiration in the *IPMDH* mutants. It has been found that a large number of enzymes involved in amino acid metabolism were Trx and GRX targets [48], and amino acid metabolism affected cellular redox homeostasis and altered the abundances of photosynthesis/photorespiration-related enzymes in soybean [49]. In this study, some enzymes involved in TCA cycle and glutamate metabolism in the mutants were increased, including citrate synthase (CS), aconitate hydratase (AH), isocitrate dehydrogenase (IDH), malate dehydrogenase (MDH), GS1, GGAT, and ArgJ (Figures 2, 4 and 6). As a result, some metabolites related to glutamate/glutathione metabolism (e.g., 2-oxo-glutarate, gamma-aminobutyric acid, and ophthalmate) were also found to be increased in the mutants (Figure 5). This indicates that the amino acid imbalance in the mutants can alter cellular respiration and other metabolic processes.

### Reduced Photosynthesis and Enhanced Oxidative Stress Caused Growth Retardation

Amino acid metabolism has been known to closely associate with photosynthesis [50], although the mechanism of coordination is not known. Perturbation of amino acid metabolism by herbicide glyphosate affected cellular redox status and the levels of photosynthesis proteins in soybean leaves [49]. In this study, we observed that the leaf biomass and chlorophyll contents in the double and triple mutants were significantly reduced (Figure 1). In addition, several photosynthesis-related proteins exhibited gene-dosage dependent decreases in the mutants. For example, two chlorophyll formation-related enzymes (protochlorophyllide oxidoreductase (POR) B and POR C), 15 PSII proteins, two PSI proteins, a post-illumination chlorophyll fluorescence increase (PIFI) protein involved in chlororespiratory electron transport, and a Calvin cycle enzyme (fructose bisphosphate aldolase) were reduced in the *IPMDH* mutants (Figures 2, 4 and 6). Furthermore, several proteins involved in membrane stability (e.g., thylakoid soluble phosphoprotein (TSP) 9, fibrillin, lumenal cyclophillin (CYP), and TLPs), proteins synthesis and folding (CRNP and CHSP70), and redox regulation (Prx and Trx) in thylakoid were all decreased in the mutants (Figures 2, 4 and 6). The reduction of photosynthesis proteins could reasonably lead to the decrease of light and “dark” reactions and thus the low biomass of the double and triple mutants.

In the mutants (especially the triple mutants), there were indications of increased protein and nucleic acid turnover, and oxidative stress. First, high levels of hydroxyproline in the mutants often indicate proteolysis [51]. Second, increased levels of pseudouridine and 2′,3′-cyclic nucleotides (2′,3′-cAMP and 2′,3′- 2′,3′-cGMP) indicated RNA turnover. These compounds were generated from degradation of tRNA splicing intermediates under stress conditions in plants [52]. Third, the mutants had higher levels of sinapate and salicylate, suggesting induction of the phenylpropanoid pathway (a response also indicative of stress), as well as higher levels of ascorbate and ophthalmate (a compound generated from the glutathione pathway during periods of high glutathione demand), both suggesting increased oxidative stress. Fourth, elevated levels of citramalate and maleate were observed in the mutant plants. Citramalate (also named 2-ethylmalate) was formed by an enzymatic reaction analogous to that of 2-isopropylmalate synthesis, by the reaction of acetyl-CoA and water with pyruvate. The compound is an early precursor for Val and Ile synthesis. The increase in citramalate could be a result of metabolic channeling due to the blockage of leucine biosynthesis (Figure 6). Maleate was an isomerization of fumarate or an oxidative dehydration product of malate. It may also be an oxidative catabolite of some larger molecules. Last but not least, the induced ROS scavenging enzymes and stress/defense-related proteins described in the results and previous sections clearly indicate cellular oxidative stress, which can affect activities of many enzymes including those in photosynthesis. Reduced photosynthesis and enhanced oxidative stress constitute major factors leading to growth retardation of the *IPMDH* mutants.

## Supporting Information

Table S1 **List of primer sequences used for the RT-PCR experiments.**
(XLS)

Table S2 **2D-DIGE experimental design and list of proteins in wild-type and the mutant plants identified and quantified using the 2D-DIGE approach.**
(XLS)

Table S3 **List of proteins in wild-type and the** ***IPMDH*** **mutant plants identified and quantified using the iTRAQ approach.**
(XLS)

Table S4 **Identification and quantification of metabolites in wild-type and the** ***IPMDH*** **mutant plants using GC-MS and UPLC-MS.**
(XLS)

Figure S1 **Co-expression analysis of glucosinolate metabolism-related genes using ATTED-II (http://atted.jp** **) (Chen et al., 2011).** The mutase was highlighted in yellow. The dots of different colors indicate different pathways that the genes are involved in. Red represents biosynthesis of secondary metabolites, yellow represents cysteine and methionine metabolism, green represents the metabolism of glycine, serine and threonine, light blue represents sulfur metabolism, and dark blue represents selenoamino acid metabolism.
(TIF)

Figure S2 **RT-PCR verification of the** ***IPMDH*** **mutants.** (A) Schematic diagram of the genomic structure of *IPMDHs* with T-DNA insertion sites (triangles) and the primers sites. Bars and lines represent exons and introns, respectively. ATG, start codon; STOP, stop codon. (Scale bar, 250 bp). (B) Semiquantitative RT-PCR analysis. The *actin* gene was used as a loading control.
(TIF)

Figure S3 **Precursor contamination in the iTRAQ experiments of wild-type (113 and 117), the single mutant (** ***aaBBDD*** **, 114 and 118), double mutant (** ***AABbdd*** **, 115 and 119), and triple mutant (** ***aaBbdd*** **, 116 and 121).** (A) Three identified peptides in the sequence of IPMDH1. (B–D) Quantitative mass spectrometry (MS) patterns of the three peptides in wild-type and the mutants as represented by intensities of the 8-plex iTRAQ tags. The red line in each peak shows MS background, and the blue line indicates real signal of the peptide. Green arrows indicate the extremely low quantitative signals from *ipmdh* single mutant after background subtraction. (E) Shotgun LC-MS results show that only peptides from IPMDH2 and IPMDH3 exist in the *ipmdh1* (*aaBBDD*) mutant, supporting that the *ipmdh1* was a knock out mutant; (F) Shotgun LC-MS peptide list shows that all of peptides from IPMDH1, IPMDH2 and IPMDH3 exist in the wide-type plants.
(TIF)

Figure S4 **3-isopropylmalate contents in different** ***Arabidopsis IPMDH*** **mutants and wide-type plants.** A, *IPMDH1*; a, *ipmdh1*; B, *IPMDH2*; b, *ipmdh2*; D, *IPMDH3*; d, *ipmdh3*. Five biological replicates of each sample were used.
(TIF)
