# Supplementary figures and images for: Integrated Proteomics and Metabolomics of Arabidopsis Acclimation to Gene-Dosage Dependent Perturbation of Isopropylmalate Dehydrogenases
Source: PLoS One. 2013 Mar 22;8(3):e57118. doi: 10.1371/journal.pone.0057118 (PMC3606340; doi:10.1371/journal.pone.0057118)

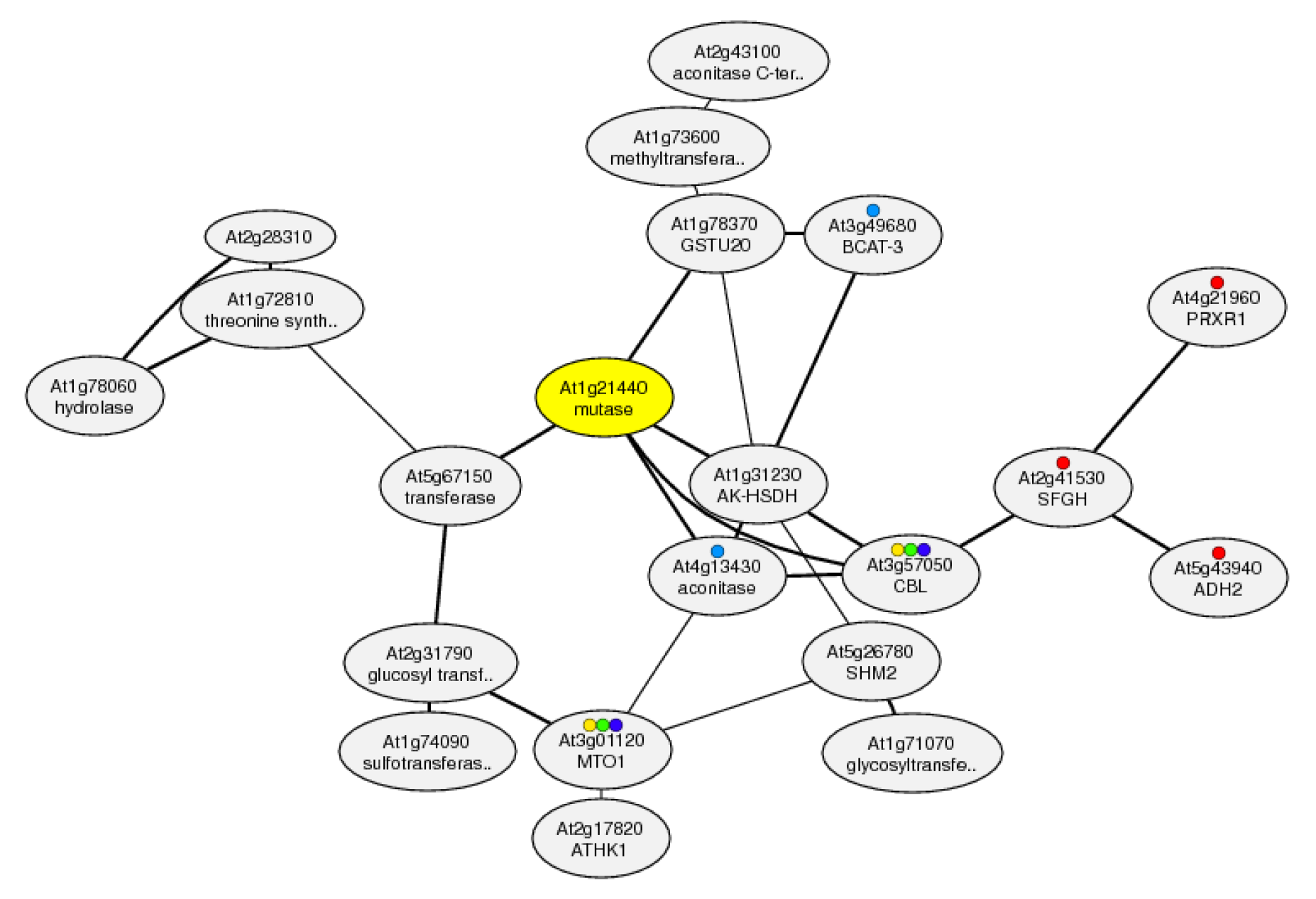

Supplement: Figure S1 — Co-expression analysis of glucosinolate metabolism-related genes using ATTED-II (http://atted.jp ) (Chen et al., 2011). The mutase was highlighted in yellow. The dots of different colors indicate different pathways that the genes are involved in. Red represents biosynthesis of secondary metabolites, yellow represents cysteine and methionine metabolism, green represents the metabolism of glycine, serine and threonine, light blue represents sulfur metabolism, and dark blue represents selenoamino acid metabolism. (TIF) [file pone.0057118.s005.tif]

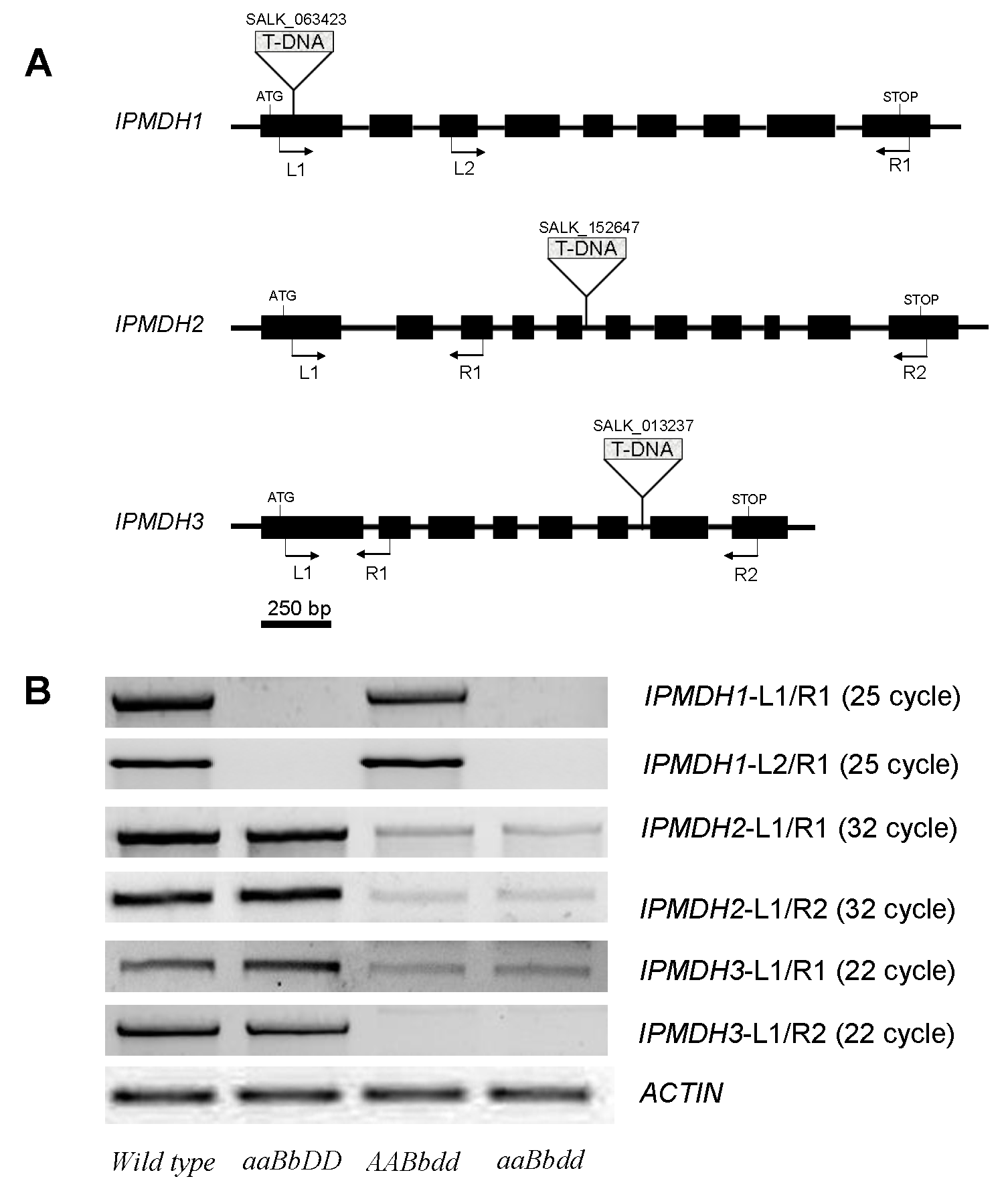

Supplement: Figure S2 — RT-PCR verification of the IPMDH mutants. (A) Schematic diagram of the genomic structure of IPMDHs with T-DNA insertion sites (triangles) and the primers sites. Bars and lines represent exons and introns, respectively. ATG, start codon; STOP, stop codon. (Scale bar, 250 bp). (B) Semiquantitative RT-PCR analysis. The actin gene was used as a loading control. (TIF) [file pone.0057118.s006.tif]

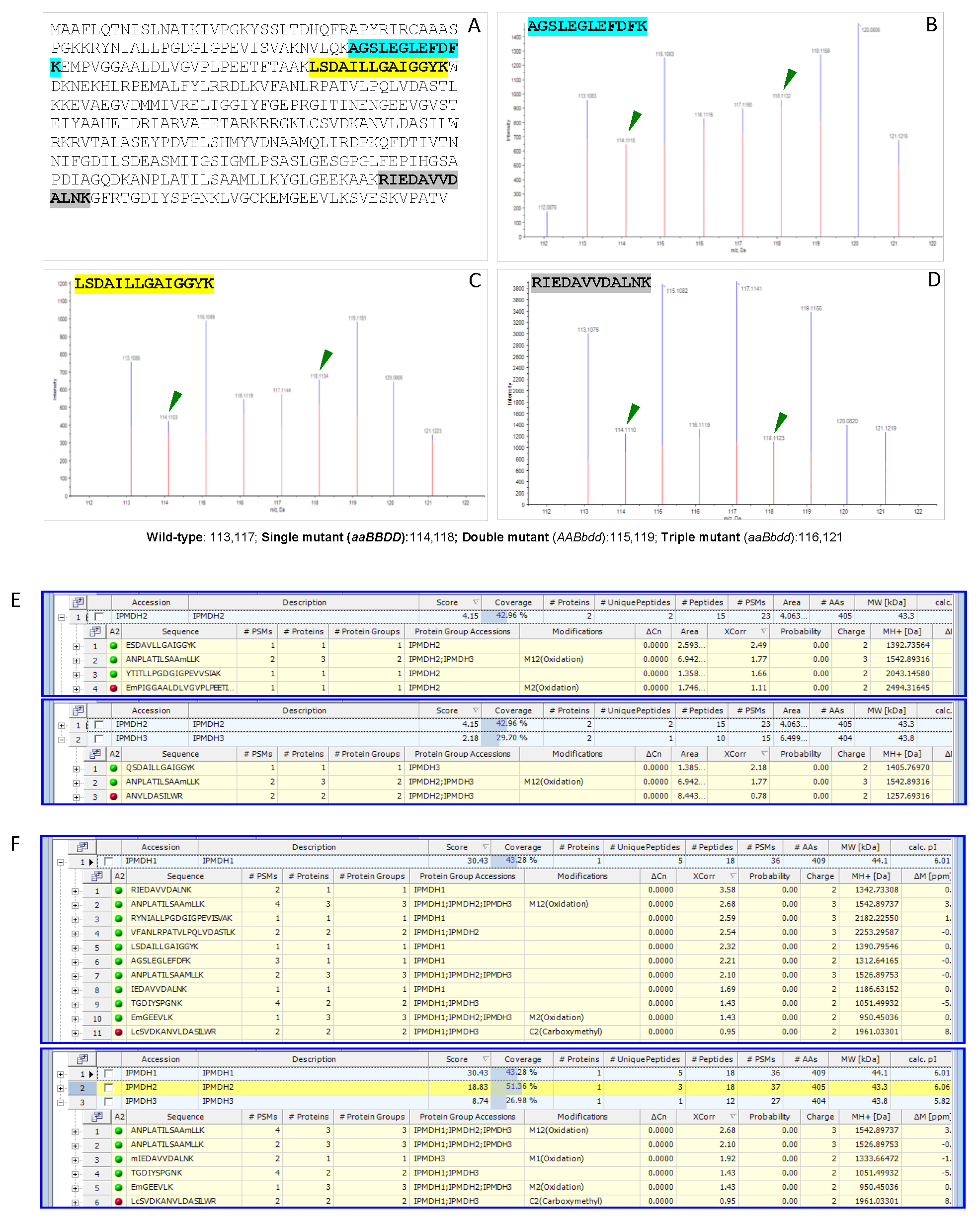

Supplement: Figure S3 — Precursor contamination in the iTRAQ experiments of wild-type (113 and 117), the single mutant ( aaBBDD , 114 and 118), double mutant ( AABbdd , 115 and 119), and triple mutant ( aaBbdd , 116 and 121). (A) Three identified peptides in the sequence of IPMDH1. (B–D) Quantitative mass spectrometry (MS) patterns of the three peptides in wild-type and the mutants as represented by intensities of the 8-plex iTRAQ tags. The red line in each peak shows MS background, and the blue line indicates real signal of the peptide. Green arrows indicate the extremely low quantitative signals from ipmdh single mutant after background subtraction. (E) Shotgun LC-MS results show that only peptides from IPMDH2 and IPMDH3 exist in the ipmdh1 (aaBBDD) mutant, supporting that the ipmdh1 was a knock out mutant; (F) Shotgun LC-MS peptide list shows that all of peptides from IPMDH1, IPMDH2 and IPMDH3 exist in the wide-type plants. (TIF) [file pone.0057118.s007.tif]

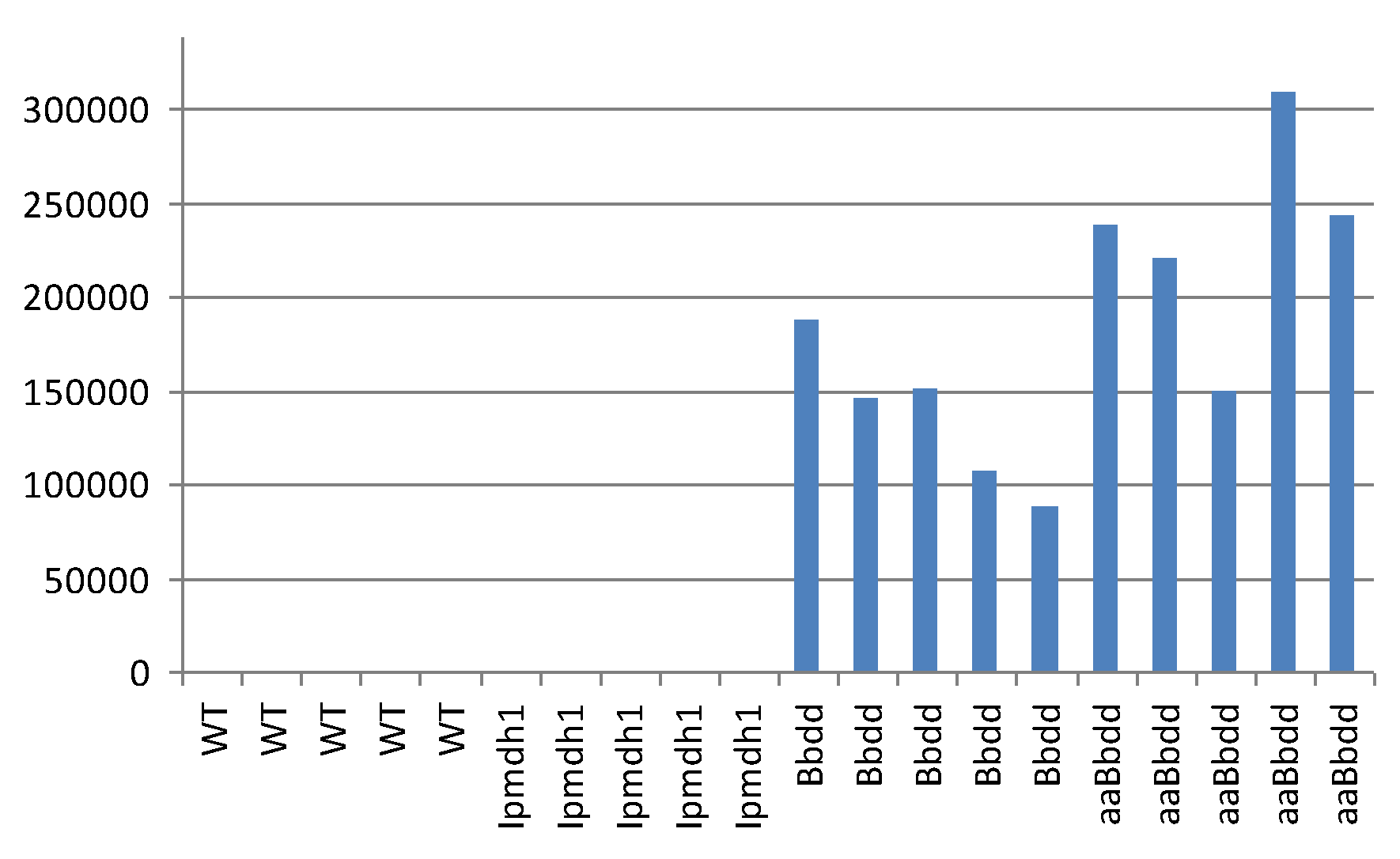

Supplement: Figure S4 — 3-isopropylmalate contents in different Arabidopsis IPMDH mutants and wide-type plants. A, IPMDH1; a, ipmdh1; B, IPMDH2; b, ipmdh2; D, IPMDH3; d, ipmdh3. Five biological replicates of each sample were used. (TIF) [file pone.0057118.s008.tif]
